# Mechanistic Insights into *Eimeria tenella*-Induced Host Cell Apoptosis Through Modulation of the Mitochondrial Permeability Transition Pore

**DOI:** 10.3390/microorganisms13092139

**Published:** 2025-09-12

**Authors:** Rui Bai, Shuying Zhu, Hui Wang, Chenyang Lv, Wenlong Zhao, Li Zhang, Yao Liu, Hanze Gao, Xiaoling Lv, Jianhui Li, Xiaozhen Cui

**Affiliations:** 1 College of Veterinary Medicine, Shanxi Agricultural University, Jinzhong 030801, China; 2 College of Animal Science, Shanxi Agricultural University, Jinzhong 030801, China

**Keywords:** *Eimeria tenella*, host cell, apoptotic, mitochondrial intermembrane protein

## Abstract

Coccidiosis due to *Eimeria tenella* remains a major constraint on the poultry industry. Previous studies have revealed that *E. tenella* infection triggers apoptosis in host cells. The mitochondrial permeability transition pore (MPTP) plays a pivotal role in the apoptosis and necrosis observed in infected host cells. However, the effect of MPTP opening on mitochondrial apoptotic factors remains unclear. To elucidate the dynamic changes in apoptotic signals during MPTP-mediated apoptosis in host cells infected with *E. tenella*, we established a chicken embryo caecal epithelial cell infection model. Cyclosporin A (CsA) was used to inhibit the MPTP. The infection rate was assessed by Hematoxylin and eosin (H&E) staining, whereas MPTP opening and the abundances of the mitochondrial apoptotic factors Smac, Endo G, and AIF were determined by flow cytometry and ELISA, respectively. The results revealed that both the degree of MPTP opening was markedly reduced in the *E. tenella+CsA* group compared to the *E. tenella* group (*p* < 0.05). Between 24 and 120 h post-infection (hpi), the cytoplasmic levels of Smac, Endo G, and AIF were significantly elevated in the *E. tenella* group compared with the control group (*p* < 0.05), while their mitochondrial levels were markedly decreased (*p* < 0.05). In contrast, mitochondrial expression of these factors was restored in the *E. tenella+CsA* group (*p* < 0.05), accompanied by a reduction in their cytoplasmic abundance (*p* < 0.05). These findings indicate that *E. tenella* promotes MPTP-dependent release of mitochondrial pro-apoptotic factors into the cytosol during the mid-to-late stages of infection, whereas pharmacological inhibition of the MPTP limits this redistribution.

## 1. Introduction

*Eimeria tenella* (*E. tenella*) is one of the most virulent coccidian species and predominantly infects the chicken caecum [1]. Infection often leads to reduced weight gain and egg production, and in severe cases, mortality can reach up to 80%, resulting in an estimated global economic loss of approximately $14.5 billion annually [2]. *E. tenella* infection causes swelling, collapse, and necrotic shedding of mucosal epithelial cells, thereby contributing to severe mucosal damage [3]. This structural damage is accompanied by increased apoptosis in caecal mucosal and intestinal gland epithelial cells [4], which represents a key pathogenic mechanism underlying host tissue injury. During early infection, expression of both anti- and pro-apoptotic genes (e.g., Bax and Bad) declines, yet the anti-/pro-apoptotic Bcl-2 ratio rises, paralleling reduced host–cell apoptosis. In contrast, in mid- to late infection, Bcl-2, Bcl-XL, Bax, Bak, Bid, Bad, and HK-II rise sharply; the anti-/pro-apoptotic ratio falls, and host–cell apoptosis surges [5].

The mitochondrion is a double-membrane-bound organelle with a sac-like structure. The compartment enclosed by the inner mitochondrial membrane is referred to as the inner chamber, while the intermembrane space between the inner and outer membranes constitutes the outer chamber [6,7]. The mitochondrial permeability transition pore (MPTP) is a multiprotein complex at inner–outer membrane contact sites, comprising structural and regulatory subunits [8,9]. Studies have demonstrated that the MPTP-induced increase in mitochondrial membrane permeability is a crucial event in mitochondrial apoptosis signaling pathways [10]. Mitochondrial apoptosis represents a major mechanism of apoptosis during the development of *E. tenella* [11]. The invasion of *E. tenella* into chicken caecal epithelial cells triggers the opening of the MPTP, reduces mitochondrial membrane potential, and enhances host cell apoptosis [12]. Cyclosporin A (CsA), a specific inhibitor of the MPTP, binds to cyclophilin D (CypD), a component of the MPTP complex, thereby preventing its opening through inhibition of peptidyl-prolyl cis-trans isomerase (PPIase) activity [13].

Mitochondria contain several key pro-apoptotic proteins, including endonuclease G (Endo G), cytochrome c (cyt c), apoptosis-inducing factor (AIF), and the second mitochondria-derived activator of caspases (Smac). Studies have shown that during the early stage of apoptosis, cyt c is released from the mitochondria into the cytoplasm, thereby triggering the mitochondrial apoptosis signaling pathway and leading to cell death [14,15]. In this pathway, Smac primarily functions by antagonizing the caspase-inhibitory activity of inhibitor of apoptosis proteins (IAPs), thereby facilitating apoptosis [16]. Additionally, upon stimulation by apoptotic signals, Endo G is released together with cyt c and AIF, two other mitochondrial apoptotic proteins. After translocating to the nucleus, Endo G mediates nuclear DNA fragmentation, ultimately leading to apoptosis [17]. Similar mitochondrial pro-apoptotic events have also been observed during infections with other pathogens. For instance, reovirus promotes the release of cyt c and Smac from the mitochondria of human embryonic kidney (HEK) cells [18]. Another study revealed the involvement of AIF and Endo G in the apoptosis of bovine mononuclear macrophages induced by *Mycobacterium* infection [19].

The intracellular levels of cyt c, Smac, Endo G, and AIF in host cells undergo significant alterations during the development of intracellular parasites, including *Toxoplasma gondii*, *Trypanosoma cruzi*, and *Leishmania* [20,21,22]. Therefore, the mechanism by which these parasites induce host cell apoptosis is believed to be intricately associated with mitochondrial apoptotic signaling pathways [23,24]. We previously demonstrated that cyt c participates in *E. tenella*-induced host–cell apoptosis via the intrinsic mitochondrial apoptotic pathway [12].

Chicken caecal epithelial cells are the primary target of *E. tenella*, yet the expression profiles of apoptotic factors in these host cells during infection remain poorly understood. Furthermore, the role of the MPTP in activating apoptotic factors during *E. tenella* infection has not been fully elucidated. This study aimed to characterize the temporal expression dynamics of mitochondrial apoptotic factors in infected host cells and to elucidate their relationship with MPTP activity. Using a chicken embryo caecal epithelial cell model, we employed hematoxylin and eosin (H&E) staining, flow cytometry, ELISA, and related assays to investigate how MPTP opening modulates the expression of key apoptotic regulators. This study aimed to elucidate the temporal dynamics of mitochondrial apoptotic factors in chicken caecal epithelial cells during *E. tenella* infection and to determine whether these events are regulated by MPTP opening, thereby providing novel insights into mitochondria-mediated host–pathogen interactions.

## 2. Materials and Methods

### 2.1. Experimental Animals

All animal experiments were conducted in accordance with the principles of laboratory animal care and the guidelines established by the Institutional Animal Care and Use Committee of Shanxi Agricultural University. Every effort was made to minimize animal suffering. The study protocol was approved by the Institutional Animal Care and Use Committee of Shanxi Agricultural University. A total of 10 1-day-old chicks and 100 15-day-old specific pathogen-free (SPF) chicken embryos were used and supplied by Beijing Boehringer Ingelheim Vital Biotechnology Co., Ltd. (Beijing, China). The 1-day-old chicks were raised in a strictly pathogen-free environment. Isolator conditions were as follows (temperature and pressure): 1–3 days, 35–36 °C and 25 Pa; 4–7 days, 32–35 °C and 25–35 Pa; 8–14 days, 29–32 °C and 35–45 Pa; and 15–30 days, 21–25 °C and 55–75 Pa.

### 2.2. Parasites

The *E. tenella* Shanxi virulent strain (EtSX01) used in this study was kindly provided by the Laboratory of Veterinary Pathology, College of Veterinary Medicine, Shanxi Agricultural University.

### 2.3. Primary Culture of Chick Embryo Caecal Cells

Chicken embryo caecal epithelial cells were isolated and cultured as previously described [4]. Briefly, 15-day-old SPF chicken embryos were surface-sterilized, and caecal tissues were dissected, then rinsed three times with double-antibiotic PBS (Solarbio, Beijing, China). Tissues were minced into ~1 mm^3^ fragments and digested with thermophilic protease (50 mg/L; Sigma-Aldrich, St. Louis, MO, USA) at 37 °C for 2 h. The digestion was neutralized with PBS and centrifuged at 344× *g* for 5 min. The resulting cell pellet was resuspended in DMEM (HyClone, Logan, UT, USA) supplemented with 2.5% FBS (Sijiqing, Hangzhou, China), EGF (0.02 µg/mL; PeproTech, Rocky Hill, NJ, USA), penicillin–streptomycin (100 U/mL), heparin (0.1 mg/mL), sodium pyruvate (1.1 mg/mL), and L-glutamine (0.5 mmol/L) (all from Solarbio). Cells were counted and incubated at 41 °C in 8% CO_2_. When the adhesion rate reached 85%, they were harvested for subsequent experiments.

### 2.4. Preparation of Sporozoites of E. tenella

The preparation of *E. tenella* sporozoites followed the method described in a previous study [25]. Ten SPF chicks were raised in isolators until 20 days of age and orally inoculated with 5 × 10^3^ sporulated *E. tenella* oocysts per chick. On days 6–8 post-infection, oocysts were collected from feces, sporulated, and centrifuged at 1490× *g* for 5 min. The supernatant was discarded, and the pellet was resuspended in 2 mL PBS. The sample was ground using a glass rod until the excystation rate reached approximately 80%. The homogenate containing sporocysts was centrifuged at 955× *g* for 10 min, and the precipitate was resuspended in an appropriate volume of sporocyst digestion solution (0.75%, *v*/*v*, trypsin from Solarbio and 10% bile from SPF chickens). The mixture was shaken and incubated at 41 °C for 70 min until ≥80% sporozoite release was achieved. After filtration, the sample was centrifuged again at 1490× *g* for 10 min, and the pellet was resuspended in infection medium for counting. The infection medium consisted of low-glucose DMEM (HyClone), MEM199 (HyClone) mixed in a 2:1 (*v*/*v*) ratio, 1.5% FBS (Sijiqing), EGF (0.02 μg/mL; PeproTech), glucose (0.3 mg/mL), penicillin (100 U/mL), streptomycin (100 U/mL), heparin (0.1 mg/mL), sodium pyruvate (1.1 mg/mL), insulin (0.05 μg/mL), L-glutamine (0.5 mM), folic acid (0.05 μg/mL), vitamin B6 (0.05 μg/mL), and vitamin B1 (0.05 μg/mL) (all from Solarbio, Beijing, China).

### 2.5. Experimental Protocol

Chick embryo caecal epithelial cells cultured in six-well plates and chamber slides were harvested at approximately 85% confluence and randomly assigned to three experimental groups: (1) the control group, (2) the *E. tenella* group (infected with *E. tenella* sporozoites), and (3) the *E. tenella+CsA* group (infected with *E. tenella* sporozoites and treated with CsA; 20 μM, Dalian Mellon Biological Technology Co., Ltd., Dalian, China). In the infected groups, cells were inoculated with 4 × 10^5^ freshly excysted *E. tenella* sporozoites per well. For the *E. tenella+CsA* group, cells sampled at 4 h post-infection (hpi) received CsA treatment at the time of infection, whereas those sampled at 24–120 hpi were treated with CsA 24 h prior to sampling. All groups were maintained for up to 120 hpi at 41 °C in a humidified incubator with 8% CO_2_, and the culture medium was refreshed every 48 h. Samples were collected at 4, 24, 48, 72, 96, and 120 hpi. Five replicates per group were used to assess infection rate, apoptosis rate, and Smac, AIF, and Endo G expression levels in both the mitochondria and the cytoplasm.

### 2.6. H&E Staining and Quantification of E. tenella Infection Rates

At 4, 24, 48, 72, 96, and 120 hpi, the infection rate was assessed in each group following H&E staining. The procedures were conducted as previously described before the study [12]. In each sample, 200 cells were randomly selected and examined under a light microscope to evaluate *E. tenella* infection. The infection rate at each time point was calculated using the following formula:

Infection rate (%) = (number of infected cells observed at each time point/200) × 100.

### 2.7. Dynamic Assessment of MPTP Opening in E. tenella-Infected Cells

The dynamic changes in MPTP opening in *E. tenella*-infected cells were evaluated by flow cytometry (FCM) using Calcein-AM (Thermo Fisher Scientific, Carlsbad, CA, USA) combined with CoCl_2_ (Sigma-Aldrich). Briefly, caecal epithelial cells from *E. tenella*-infected chicken embryos were harvested and suspended in 200 μL of loading buffer. The cells were stained with Calcein-AM and incubated at 37 °C for 15 min, followed by washing with Hank’s balanced salt solution. Next, the cells were treated with CoCl_2_ at 37 °C for 10 min. After adding 250 μL of binding buffer, the samples were analyzed by flow cytometry. The fluorescence intensity of Calcein-AM reflects the extent of MPTP opening, with higher fluorescence indicating a lower degree of pore opening [8].

### 2.8. Mitochondrial Isolation and Dynamic Detection of Apoptotic Factor Levels in E. tenella-Infected Host Cells

Mitochondria were isolated from *E. tenella*-infected chick embryo caecal epithelial cells using a mitochondrial isolation kit (Beyotime, Shanghai, China), following the manufacturer’s instructions. The dynamic changes in the levels of cyt c, Smac, Endo G, and AIF in whole-cell lysates, mitochondrial fractions, and cytoplasmic fractions at various time points post-infection were quantified using ELISA kits specific for Smac, Endo G, and AIF (all from MyBioSource, San Diego, CA, USA), according to the manufacturers’ protocols.

### 2.9. Statistical Analysis

All quantitative data are expressed as mean ± standard deviation (SD) and were analyzed using SPSS version 22.0 (IBM Corp., Armonk, NY, USA). One-way analysis of variance (ANOVA) followed by Tukey’s post hoc test was employed to determine statistical significance among experimental groups. Data visualization was performed using GraphPad Prism version 5.0 (GraphPad Software, San Diego, CA, USA). Each experiment was repeated at least three times. Differences were considered statistically significant at *p* < 0.05 and highly significant at *p* < 0.01.

## 3. Results

### 3.1. Inhibition of MPTP Selectively Enhances Intracellular Expansion of E. tenella

**Figure 1:**
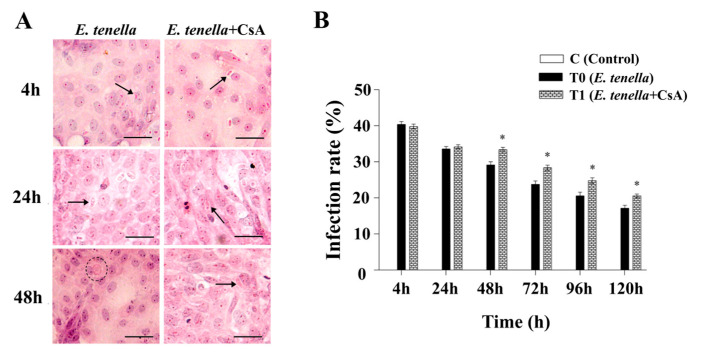
*E. tenella* infection rates. (**A**) In vitro development of *E. tenella* in each group as visualized by haematoxylin and eosin staining. Sporozoites (arrows) and trophozoites (circles) are marked accordingly. Scale bar = 20 μm. (**B**) Quantification of *E. tenella* infection rates in host cells under different treatments. C = uninfected control (infection rate = 0% at all time points; displayed as a zero-height bar. Notes: Data are presented as mean ± SD (*n* = 3). “*” indicates a statistically significant difference between the *E. tenella+CsA* group and the *E. tenella*-infected group (*p* < 0.05).

H&E staining revealed the presence of intracellular *E. tenella* in both groups, with a progressive increase in parasite burden over time. At 4 and 24 hpi, there were no statistically significant differences in infection rates between the *E. tenella* group and the *E. tenella+CsA* group (*p* > 0.05). However, starting at 48 hpi, the *E. tenella+CsA* group exhibited a significantly greater parasite burden in epithelial cells compared to the *E. tenella* group (*p* < 0.05, Figure 1A,B). Quantitative analysis of infection rates further confirmed that between 48 and 120 hpi, the infection rate in the *E. tenella+CsA* group was significantly higher than that in the *E. tenella* group (*p* < 0.05; Figure 1B). These results suggest that inhibiting MPTP opening does not affect the early invasion of *E. tenella* but enhances its intracellular replication and development at later stages of infection.

### 3.2. CsA Preserves Mitochondrial Integrity by Inhibiting E. tenella-Induced MPTP Opening

**Figure 2:**
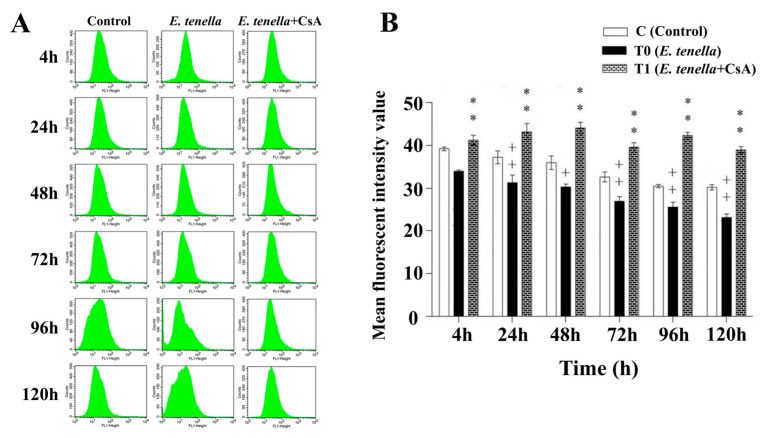
Effects of *E. tenella* infection and CsA treatment on MPTP opening in chick embryo caecal epithelial cells. (**A**) Representative flow cytometry histograms showing changes in MPTP opening at different time points (4, 24, 48, 72, 96, and 120 hpi). (**B**) Quantification of MFI of calcein-AM at each time point. Notes: Data are presented as mean ± SD (*n* = 3). “**” indicates a highly significant difference between the *E. tenella+CsA* group and the *E. tenella*-infected group (*p* < 0.01). “+” indicates a statistically significant difference between the *E. tenella*-infected group and the control group (*p* < 0.05); “++” indicates a highly significant difference (*p* < 0.01). The same significance annotations apply throughout the manuscript.

Flow cytometric analysis using the Calcein-AM/CoCl_2_ fluorescence quenching assay revealed a progressive decline in mitochondrial mean fluorescence intensity (MFI) in *E. tenella*-infected cells between 4 and 120 hpi, accompanied by a gradual leftward shift in the fluorescence peak (Figure 2A). Quantitative data showed that the mitochondrial MFI in the *E. tenella*-infected group remained consistently lower than that in the control group at all examined time points, with a statistically significant reduction observed from 24 to 120 hpi (*p* < 0.01; Figure 2B). In contrast, post-infection CsA treatment helped preserve mitochondrial fluorescence, as indicated by significantly higher calcein-AM fluorescence intensity compared to the *E. tenella*-infected group (*p* < 0.01; Figure 2B). These findings suggest that *E. tenella* induces rapid opening of the MPTP in host intestinal epithelial cells, which progressively intensifies throughout the course of infection, whereas CsA effectively inhibits MPTP opening and preserves mitochondrial integrity.

### 3.3. Temporal MPTP-Driven Smac Redistribution During E. tenella Infection

**Figure 3:**
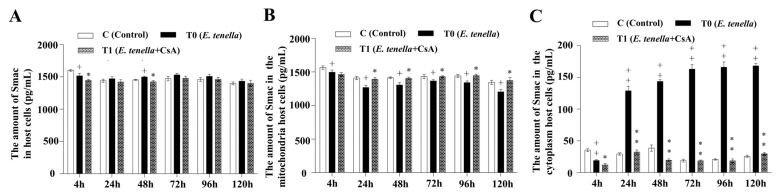
The dynamic changes in Smac levels in *E. tenella*-infected chicken embryo cecal epithelial cells, measured by ELISA (pg/mL). (**A**) Effects of *E. tenella* on total Smac in chicken embryo cecal epithelial cells. (**B**) Effects of *E. tenella* on mitochondrial Smac in chicken embryo cecal epithelial cells. (**C**) Effects of *E. tenella* on cytoplasmic Smac in chicken embryo cecal epithelial cells. Notes: Data are presented as mean ± SD (*n* = 3). “*” indicates a statistically significant difference between the *E. tenella* + CsA group and the *E. tenella*-infected group (*p* < 0.05); “**” indicates a highly significant difference (*p* < 0.01). “+” indicates a statistically significant difference between the *E. tenella*-infected group and the control group (*p* < 0.05); “++” indicates a highly significant difference (*p* < 0.01).

The expression of Smac protein in the *E. tenella*-infected group was significantly decreased at 4 hpi, but markedly increased at 48 hpi compared to the control group (*p* < 0.05). At both 4 and 48 hpi, Smac levels were markedly lower in the *E. tenella+CsA* group compared to the *E. tenella* group (*p* < 0.05; Figure 3A). In mitochondrial fractions, Smac expression in the *E. tenella* group was significantly lower than that in the control group throughout 4–120 hpi (*p* < 0.05), while the *E. tenella+CsA* group exhibited elevated levels of mitochondrial Smac compared to the *E. tenella* group from 24 to 120 hpi (*p* < 0.05; Figure 3B). A significant reduction in cytoplasmic Smac levels was observed in the *E. tenella* group at 4 hpi (*p* < 0.01), followed by a marked increase from 24 to 120 hpi compared to the control (*p* < 0.01). Moreover, cytoplasmic Smac levels in the *E. tenella+CsA* group were significantly lower than those in the *E. tenella* group at all examined time points (*p* < 0.05; Figure 3C). These findings indicate that Smac expression is regulated through MPTP opening during the middle and late stages of *E. tenella* infection.

### 3.4. Temporal MPTP-Driven Endo G Redistribution During E. tenella Infection

**Figure 4:**
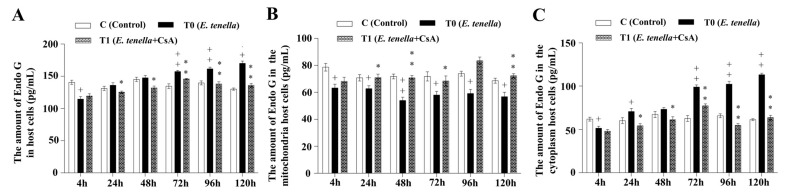
The dynamic changes in Endo G levels in *E. tenella*-infected chicken embryo cecal epithelial cells, measured by ELISA (pg/mL). (**A**) Effects of *E. tenella* on total Endo G in chicken embryo cecal epithelial cells. (**B**) Effects of *E. tenella* on mitochondrial Endo G in chicken embryo cecal epithelial cells. (**C**) Effects of *E. tenella* on cytoplasmic Endo G in chicken embryo cecal epithelial cells. Notes: Data are presented as mean ± SD (*n* = 3). “*” indicates a statistically significant difference between the *E. tenella* + CsA group and the *E. tenella*-infected group (*p* < 0.05); “**” indicates a highly significant difference (*p* < 0.01). “+” indicates a statistically significant difference between the *E. tenella*-infected group and the control group (*p* < 0.05); “++” indicates a highly significant difference (*p* < 0.01).

The total protein expression of Endo G in host cells was significantly reduced in the *E. tenella*-infected group at 4 hpi (*p* < 0.05), but significantly increased at 72, 96, and 120 hpi compared to the control group (*p* < 0.01). The total Endo G expression level in the *E. tenella+CsA* group was significantly lower than that in the *E. tenella* group from 24 to 120 hpi (*p* < 0.05; Figure 4A). In mitochondrial fractions, Endo G expression was significantly lower in the *E. tenella* group than in the control group from 4 to 120 hpi (*p* < 0.05), while the *E. tenella+CsA* group exhibited significantly higher mitochondrial Endo G levels compared to the *E. tenella* group during the same period (*p* < 0.05; Figure 4B). In the cytoplasmic fraction, Endo G levels were significantly reduced in the *E. tenella* group at 4 hpi (*p* < 0.05), but significantly elevated at 24, 72, 96, and 120 hpi relative to the control (*p* < 0.05). Moreover, cytoplasmic Endo G expression in the *E. tenella+CsA* group was consistently lower than that in the *E. tenella* group from 24 to 120 hpi (*p* < 0.05; Figure 4C). These results indicate that Endo G expression is regulated by MPTP opening during the middle and late stages of *E. tenella* infection.

### 3.5. Temporal MPTP-Driven AIF Redistribution During E. tenella Infection

**Figure 5:**
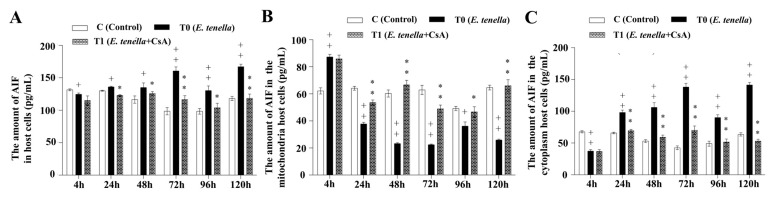
The dynamic changes in AIF levels in *E. tenella*-infected chicken embryo cecal epithelial cells, measured by ELISA (pg/mL). (**A**) Effects of *E. tenella* on total AIF in chicken embryo cecal epithelial cells. (**B**) Effects of *E. tenella* on mitochondrial AIF in chicken embryo cecal epithelial cells. (**C**) Effects of *E. tenella* on cytoplasmic AIF in chicken embryo cecal epithelial cells. Notes: Data are presented as mean ± SD (*n* = 3). “*” indicates a statistically significant difference between the *E. tenella* + CsA group and the *E. tenella*-infected group (*p* < 0.05); “**” indicates a highly significant difference (*p* < 0.01). “+” indicates a statistically significant difference between the *E. tenella*-infected group and the control group (*p* < 0.05); “++” indicates a highly significant difference (*p* < 0.01).

The expression of AIF in host cells was significantly lower in the *E. tenella* group than in the control group at 4 hpi (*p* < 0.05), but significantly higher from 24 to 120 hpi (*p* < 0.05). AIF expression levels in the *E. tenella+CsA* group were significantly reduced compared to the *E. tenella* group throughout the 24–120 hpi period (*p* < 0.05; Figure 5A). In mitochondrial fractions, AIF expression was significantly elevated in the *E. tenella* group at 4 hpi (*p* < 0.01), but significantly decreased from 24 to 120 hpi compared to controls (*p* < 0.01). Notably, mitochondrial AIF levels were significantly higher in the *E. tenella+CsA* group than in the *E. tenella* group during this same period (*p* < 0.01; Figure 5B). In the cytoplasm, AIF expression in the *E. tenella* group was significantly lower than that in the control group at 4 hpi (*p* < 0.01), but significantly higher from 24 to 120 hpi (*p* < 0.01). Cytoplasmic AIF levels in the *E. tenella+CsA* group remained significantly lower than those in the *E. tenella* group at all corresponding time points (*p* < 0.01; Figure 5C). These results indicate that AIF expression is regulated by MPTP opening during the middle and late stages of *E. tenella* infection.

## 4. Discussion

In this study, we investigated the mitochondrial apoptotic signaling pathways in chicken embryonic caecal epithelial cells infected with *E. tenella*. The results demonstrate that apoptosis is primarily mediated by the opening of the MPTP and the mitochondrial release of pro-apoptotic factors (Smac, Endo G, AIF). This finding is consistent with both our prior evidence of an increase in apoptosis during the mid-to-late stages of infection and the involvement of cyt c in the intrinsic mitochondrial apoptotic pathway [12]. These data provide important insights into the mechanism underlying *E. tenella* infection in chickens.

Mitochondria serve as central regulators of apoptosis [26,27]. Upon initiation of apoptosis in host cells, the MPTP opens, facilitating the release of cyt c and Smac into the cytoplasm. These molecules subsequently activate caspases, triggering the apoptotic cascade [28,29]. In addition, a caspase-independent apoptotic pathway has also been identified within the mitochondrial network [30]. In this pathway, AIF and Endo G exit mitochondria and enter the cytoplasm, then migrate to the nucleus. They trigger chromatin condensation and DNA fragmentation, culminating in apoptosis [31,32].

At 4 hpi, the MPTP opening in the *E. tenella*-infected group was greater than that in the control group, whereas the opening in the *E. tenella+CsA* group was reduced relative to the *E. tenella* group. Interestingly, this pattern did not correspond to the changes observed in early apoptosis, late apoptosis, and necrosis rates in host cells [12]. This discrepancy may be attributed to the involvement of multiple apoptotic signaling pathways within host cells [33]. Previous studies have shown that early *E. tenella* infection activates Akt, which lowers Bad, elevates Bcl-2, and restricts cytochrome c release. Collectively, these changes blunt host–cell apoptosis [34]. Moreover, it has been reported that Akt activation suppresses the generation and cytoplasmic release of AIF, Smac, and Endo G, thus interrupting the apoptotic cascade [35,36]. Therefore, the precise molecular mechanisms by which *E. tenella* modulates early-stage host cell apoptosis remain incompletely understood and merit further investigation.

From 24 to 120 hpi, the opening of the MPTP, the Smac, Endo G, and AIF, as well as the early apoptosis, late apoptosis, and necrosis rates, were all significantly higher in the *E. tenella*-infected group compared to the control group [12]. In contrast, the mitochondrial levels of Smac, Endo G, and AIF were significantly reduced in the *E. tenella* group. These findings indicate that *E. tenella* promotes host cell apoptosis during the middle and late stages of infection by enhancing MPTP opening and facilitating the release of pro-apoptotic factors from the mitochondria. Notably, CsA markedly reduced the cytoplasmic levels of Smac, Endo G, and AIF in infected cells. These results suggest that MPTP opening plays a critical role in regulating the translocation of mitochondrial apoptotic factors into the cytoplasm, thereby promoting apoptosis in host cells during the later stages of *E. tenella* infection.

In addition, following cadmium treatment, the mitochondrial levels of cyt c in hepatocytes are markedly reduced, while its cytoplasmic expression increases, thereby promoting apoptosis [37]. During apoptosis, Smac is also released into the cytoplasm, where it antagonizes inhibitor of apoptosis proteins (IAPs), facilitating caspase activation [38]. Similarly, pyrroloquinoline quinone translocates Endo G and AIF from mitochondria to the cytoplasm in chondrosarcoma cells, thereby heightening apoptosis [39]. These findings are consistent with our observations and further support the hypothesis that mitochondrial release of pro-apoptotic factors is a key event in the execution of apoptosis [40,41].

In line with our finding that *E. tenella* modulates host mitochondrial apoptosis through the MPTP, other apicomplexans similarly adjust host cell–death pathways. For example, HSP70 secreted by *T. gondii* binds AIF and APAF-1, elevates Bcl-2, and blocks AIF translocation and cytochrome c release, thereby curbing host mitochondrial apoptosis [23]. During its hepatic phase, *Plasmodium* rewires host metabolic and autophagic circuits to sustain cell viability and postpone apoptosis, thereby securing its own development [42]. *Cryptosporidium parvum* first activates NF-κB and elevates Bcl-2/Survivin to block apoptosis, then later induces pro-apoptotic genes and cell death, representing a stage-dependent biphasic switch [43]. Together, these studies show that apicomplexan parasites establish infection and evade host immunity by finely tuning host apoptotic pathways.

During the middle and late stages of *E. tenella* infection, host cell apoptosis was significantly elevated [12], characterized by reduced mitochondrial levels and increased cytoplasmic levels of cyt c, Smac, Endo G, and AIF. These results demonstrate that *E. tenella* promotes apoptosis by triggering MPTP opening and facilitating the translocation of mitochondrial apoptotic factors into the cytoplasm. Chicken embryonic cecal epithelial cells offer a controlled in vitro window on early *E. tenella*-host interactions, but they cannot recapitulate the immune complexity, microbiota, or mucosal milieu of the living cecum. Accordingly, the timing and function of MPTP opening in parasite growth and tissue injury should be confirmed in chick infection models to gauge the translational promise of MPTP-directed therapies. Despite these limitations, this study elucidates a key mitochondrial pathway involved in *E. tenella*-induced host cell apoptosis and provides a mechanistic basis for the development of targeted anticoccidial strategies.

## 5. Conclusions

In summary, we identify the MPTP as a central regulatory node exploited by *E. tenella* to manipulate host caecal epithelial cell fate. Parasite-induced MPTP opening orchestrates the coordinated release of Smac, Endo G, and AIF, thereby driving apoptosis. Conversely, pharmacological inhibition of MPTP by cyclosporin A preserves mitochondrial integrity but paradoxically supports deeper intracellular parasitism. By reframing host mitochondria as an active battleground rather than passive targets, our findings highlight MPTP modulation as a promising therapeutic strategy to both mitigate host tissue damage and impair parasite fitness.

## Data Availability

The original contributions presented in this study are included in the article. Further inquiries can be directed to the corresponding author.
